# Effect of progesterone on Smad signaling and TGF-β/Smad-regulated genes in lung epithelial cells

**DOI:** 10.1371/journal.pone.0200661

**Published:** 2018-07-12

**Authors:** Steffen Kunzmann, Barbara Ottensmeier, Christian P. Speer, Markus Fehrholz

**Affiliations:** 1 Clinic of Neonatology, Buergerhospital Frankfurt am Main, Frankfurt am Main, Germany; 2 University Children’s Hospital, University of Wuerzburg, Wuerzburg, Germany; University of Giessen Lung Center, GERMANY

## Abstract

The effect of endogenous progesterone and/or exogenous pre- or postnatal progesterone application on lung function of preterm infants is poorly defined. While prenatal progesterone substitution may prevent preterm birth, *in vitro* and *in vivo* data suggest a benefit of postnatal progesterone replacement on the incidence and severity of bronchopulmonary dysplasia (BPD). However, the molecular mechanisms responsible for progesterone’s effects are undefined. Numerous factors are involved in lung development, airway inflammation, and airway remodeling: the transforming growth factor beta (TGF-β)/mothers against decapentaplegic homolog (Smad) signaling pathway and TGF-β-regulated genes, such as connective tissue growth factor (*CTGF*), transgelin (*TAGLN*), and plasminogen activator inhibitor-1 (*PAI-1*). These processes contribute to the development of BPD. The aim of the present study was to clarify whether progesterone could affect TGF-β1-activated Smad signaling and CTGF/transgelin/PAI-1 expression in lung epithelial cells. The pharmacological effect of progesterone on Smad signaling was investigated using a TGF-β1-inducible luciferase reporter and western blotting analysis of phosphorylated Smad2/3 in A549 lung epithelial cells. The regulation of CTGF, transgelin, and PAI-1 expression by progesterone was studied using a promoter-based luciferase reporter, quantitative real-time PCR, and western blotting in the same cell line. While progesterone alone had no direct effect on Smad signaling in lung epithelial cells, it dose-dependently inhibited TGF-β1-induced Smad3 phosphorylation, as shown by luciferase assays and western blotting analysis. Progesterone also antagonized the TGF-β1/Smad-induced upregulation of CTGF, transgelin, and PAI-1 at the promoter, mRNA, and/or protein levels. The present study highlights possible new molecular mechanisms involving progesterone, including inhibition of TGF-β1-activated Smad signaling and TGF-β1-regulated genes involved in BPD pathogenesis, which are likely to attenuate the development of BPD by inhibiting TGF-β1-mediated airway remodeling. Understanding these mechanisms might help to explain the effects of pre- or postnatal application of progesterone on lung diseases of preterm infants.

## Background

The steroid progesterone is one of the most important hormones which maintain pregnancy [1]. Positive local effects of progesterone are induced on the myometrium, the cervix, and the local immune system, all of which prevent preterm birth [1].

In addition, progesterone reduces neonatal morbidity and morbidity/mortality of preterm infants, lowers the incidence of respiratory distress syndrome, and reduces the need for mechanical ventilation and intensive care unit admissions [2]. Moreover, replacement of estradiol and progesterone in preterm infants tailored to maintain high intra uterine estradiol and progesterone levels is associated with tendency towards a reduced incidence of bronchopulmonary dysplasia (BPD) [3, 4].

BPD remains a major challenge for preterm infants [5, 6]. This chronic lung disease is characterized by a disruption of normal lung development, leading to fewer but larger alveoli, and a simplification of lung vessels [5, 6]. BPD is multifactorial, combining extreme lung immaturity with lung injury which implicate inflammatory and remodeling reactions evoked by mechanical ventilation, oxygen stress, and/or infection [5, 6].

The transforming growth factor beta (TGF-β) / mothers against decapentaplegic homolog (Smad) signaling pathway is a key pathway involved in lung development as well as airway inflammation and airway remodeling [7] which all contribute to the development of BPD [5, 6]. Elevated expression of TGF-β and an activation of Smad signaling has been described in BPD [8–10]. Connective tissue growth factor (CTGF), a downstream mediator of TGF-β, and transgelin, which is binding and stabilizing the actin cytoskeleton, are regulated by TGF-β1 and play an important role in airway/vascular remodeling. Both are implicated in the pathogenesis of BPD [10–13]. In addition, the coagulation cascade with intraalveolar fibrin deposition is an important feature of many pulmonary diseases, and the plasminogen activator/plasmin system plays an important role in extracellular matrix deposition leading to fibrosis [14]. The level of plasminogen activator inhibitor-1 (PAI-1) is increased in BPD and respiratory distress syndrome in preterm infants [8, 9].

While positive local effects of progesterone on the cervix and the myometrium are well described, possible systemic and protective effects of progesterone on the development of BPD remain poorly understood. The aim of the present study was to clarify whether progesterone could affect TGF-β1-activated Smad signaling and CTGF/transgelin expression in lung epithelial cells. An understanding of these mechanisms might help to explain the potential protective effects of progesterone on the development of BPD.

## Methods

### Reagents

Progesterone was purchased from Sigma-Aldrich (St. Louis, MN, USA). Recombinant TGF-β1 was obtained from R&D Systems (Bio-Techne, Minneapolis, MN, USA).

### Cells

A549 cells, a human lung carcinoma cell line with characteristics of human alveolar basal epithelial cells, were purchased from ATCC (LGC Standards, Teddington, UK) [15]. A549 cells were cultured in Dulbecco’s modified Eagle’s Medium (Sigma-Aldrich) with additional 10% fetal bovine serum (Gibco, Thermo Fisher Scientific, Waltham, MA, USA), 100 U/mL penicillin, and 100 μg/mL streptomycin (Sigma-Aldrich). Experiments with TGF-β1 were performed in serum-free medium. Incubation of all cells was carried out at 37°C in a humidified atmosphere with 5% CO_2_.

### Transfection and promoter assays

The human transgelin (*TAGLN*) promoter sequence (GenBank ID EF153019.1) was cloned into the pGL3 Basic vector (Promega, Fitchburg, WI, USA) between the BglII and HindIII sites as described [16]. Cloning of the (CAGA)_12_-luciferase plasmid was described previously [17].

The TGF-β1-sensitive *PAI-1* promoter luciferase reporter construct was stably transfected into mink lung epithelial cells (MLECs) as described previously [18].

The CAGA elements were originally found in the promoter region of *PAI-1* and are activated after binding of the Smad3/4 complexes and after TGF-β1 binding to the TGF-β receptor [17]. Transfection of (CAGA)_12_-luc (2 μg) or the transgelin-promoter vector (2 μg), and Renilla luciferase control reporter vector (phRL-TK; 5 ng) into A549 cells, and measurement of luciferase activity has been described previously [16]. Results are shown as the relative increase in luminescence compared with that of the controls. Experiments were carried out in triplicate and repeated at least three times.

### RNA extraction and RT-PCR

For RNA extraction, 3 × 10^5^ A549 cells were seeded on 6-well plates (Greiner). Twenty-four hours later, the cells were washed with Dulbecco’s phosphate-buffered saline (DPBS) and treated as indicated. After the appropriate time, cells were washed again and total RNA was isolated using a NucleoSpin® RNA Kit (Macherey-Nagel, Dueren, Germany) according to the manufacturer’s protocol. Total RNA was eluted in 60 μL of nuclease-free H_2_O and stored at −80°C until reverse transcription. For reverse transcription polymerase chain reaction (RT-PCR), 1 μg of total RNA was reverse transcribed using High Capacity cDNA Reverse Transcription Kit (Applied Biosystems, Thermo Fisher Scientific) according to the manufacturer’s instructions. Upon analysis, first strand cDNA was stored at −20°C.

### Quantitative RT-PCR (qPCR)

To detect human *CTGF*, *TAGLN*, and *GAPDH* (glyceraldehyde-3-phosphate dehydrogenase) mRNA, cDNA was analyzed using 12.5 μL iQ™ SYBR® Green Supermix (Bio-Rad Laboratories, Hercules, CA, USA), 0.5 μL deionized H_2_O, and 10 pmol of each forward and reverse primer, respectively.

Primers for *CTGF*, *TAGLN*, and *GAPDH* mRNA were hCTGFfwd 5′-ACCCAACTATGATTAGAGCC-3′, hCTGFrev 5′-TTGCCCTTCTTAATGTTCTC-3′, hTAGLNfwd 5′-CGAGAAGAAGTATGACGAGG-3′, hTAGLNrev 5′-CTTGCTCAGAATCACGCC-3′, hGAPDHfwd 5′-CAAAGTTGTCATGGATGACC-3′, and hGAPDHrev 5′-CCATGGAGAAGGCTGGGG-3′, respectively. qPCR was performed on an ABI Prism 7500 Sequence Detection System (*Taq*Man®) as described previously [19]. Melt curve analyses were performed to verify single PCR products. mRNA levels were normalized to the level of *GAPDH* mRNA and mean fold changes were calculated by the ΔΔC_T_ method [20].

### Western blotting analysis

Immunoblotting was performed as described [21]. In brief, equal amounts of cellular protein were separated using SDS-PAGE, electrophoretically transferred to polyvinylidene difluoride blotting membranes (Amersham Pharmacia Biotech, Piscataway, NJ, USA). Membranes were blocked in 5% bovine serum albumin and incubated with primary antibodies recognizing CTGF (ab6992; Abcam, Cambridge, UK), transgelin (sc-50446; Santa Cruz Biotechnology, Santa Cruz, CA), anti-Smad2/3-P (kind gift from Dr. C.-H. Heldin, Ludwig Institute for Cancer Research, Uppsala, Sweden), and β-actin (926–42212; LI-COR Inc., Lincoln, NE, USA), followed by staining with an horseradish peroxidase conjugated goat anti rabbit IgG (Thermo Fisher Scientific). Specific protein bands were visualized using a ChemiDoc™ MP Imaging System (Bio-Rad Laboratories, Hercules, CA). Captured signals were quantified by densitometric analysis using Image Lab™ Software v5.2.1 (Bio-Rad Laboratories).

### Data analysis

Results are given as means ± SD. Data were analyzed using one-way analysis of variance (ANOVA) with Sidak’s multiple comparisons test. A p-value ≤ 0.05 was considered statistically significant. All statistical analyses were performed using Prism® version 6 (GraphPad Software, San Diego, CA, USA).

## Results

### Effect of progesterone on Smad signaling in lung epithelial cells

**Fig 1:**
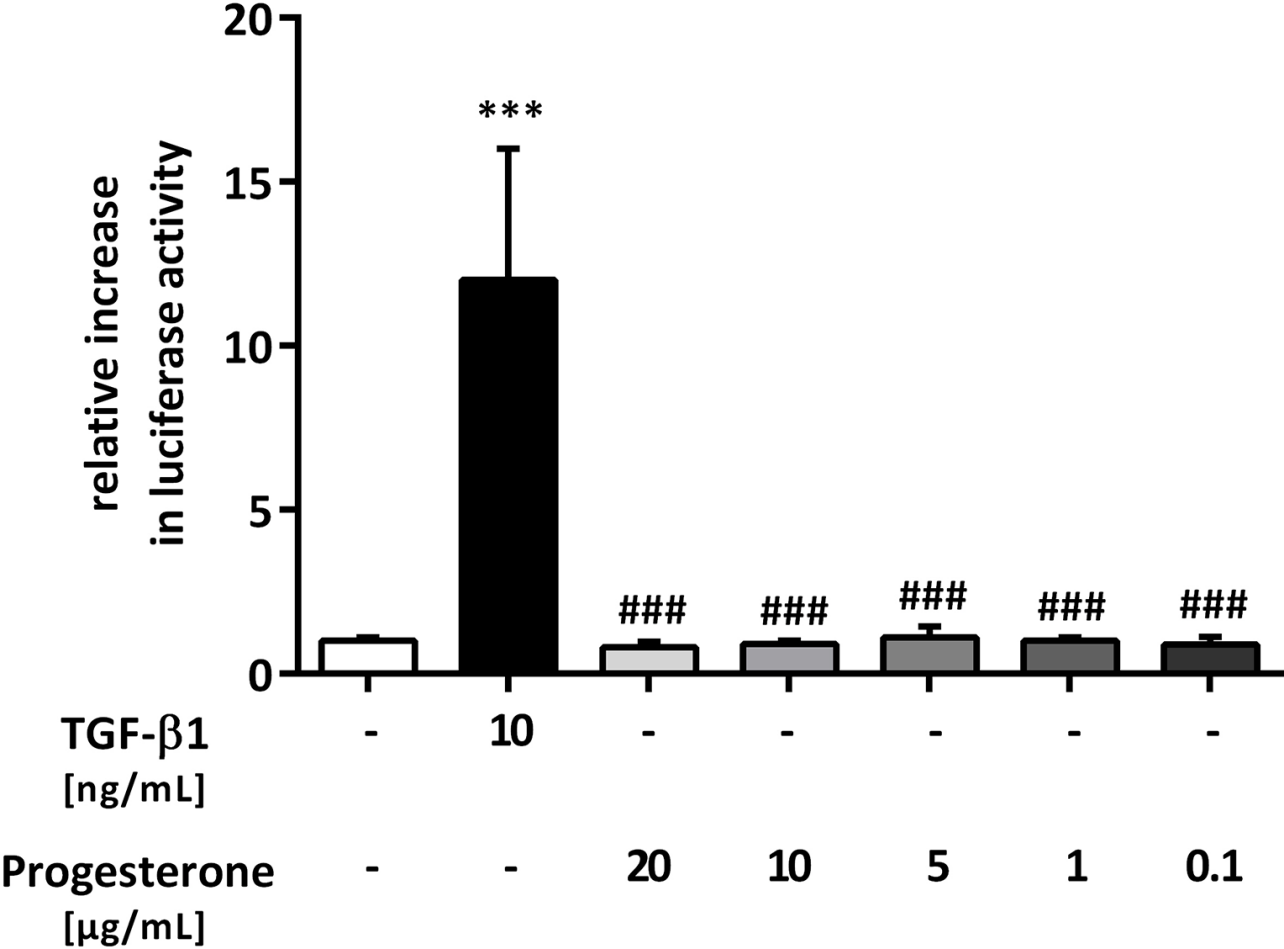
Progesterone alone does not affect Smad signaling in lung epithelial cells. The transforming growth factor beta 1 (TGF-β1)-sensitive (CAGA)_12_-luciferase reporter construct was transiently transfected into A549 cells, and the cells were treated with TGF-β1 (10 ng/mL) or with different concentrations of progesterone. Firefly luciferase activity was normalized to the activity of Renilla luciferase under control of the thymidine kinase promoter. Relative luciferase activity compared with that in the controls is shown. *** p < 0.001 compared with control cells; ### p < 0.001 compared with cells treated with TGF-β1.

**Fig 2:**
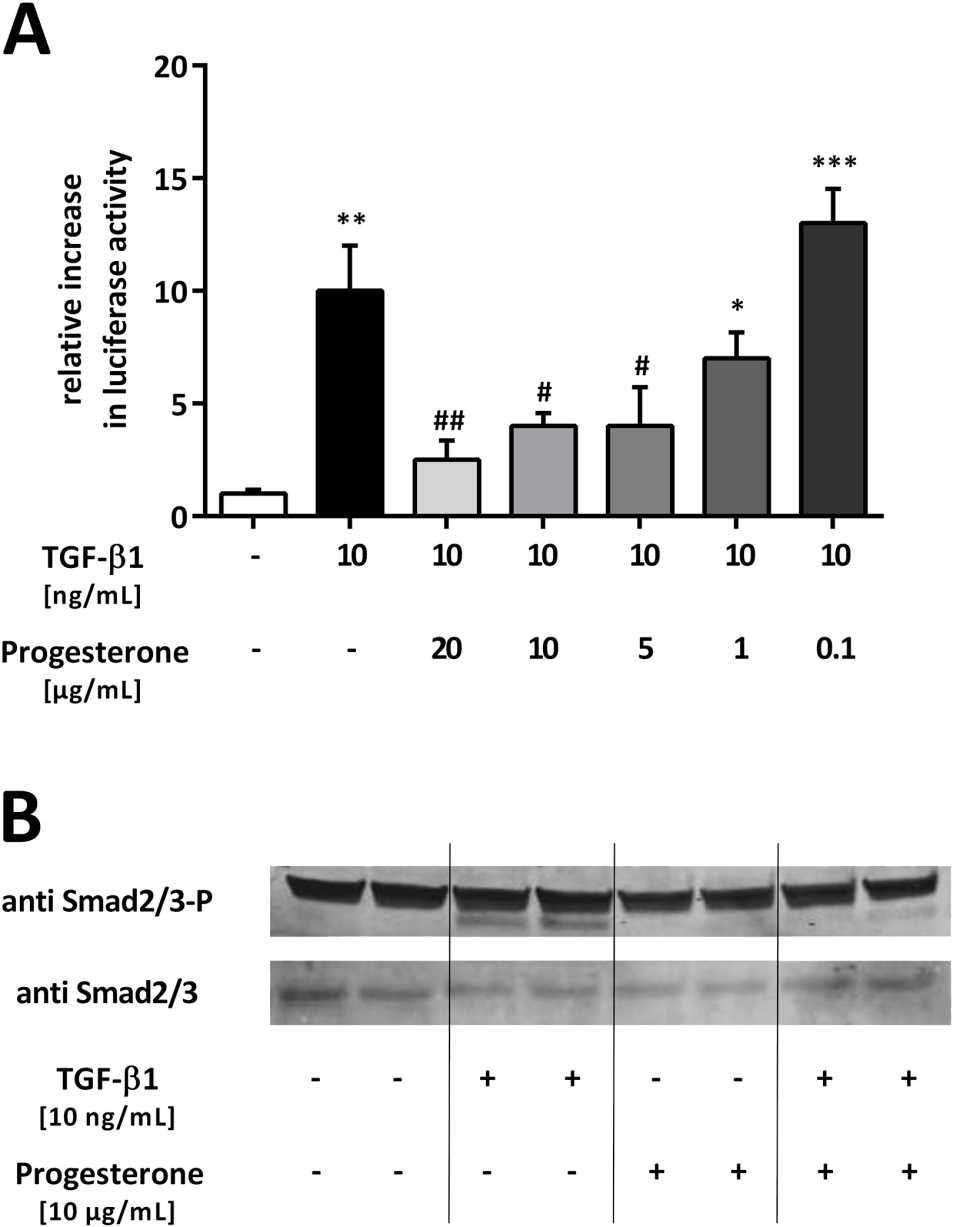
Progesterone inhibits transforming growth factor beta 1 (TGF-β1)-induced Smad signaling in lung epithelial cells. (**A**) The TGF-β1–sensitive (CAGA)_12_-luciferase reporter construct was transiently transfected into A549 cells. Cells were treated with TGF-β1 (10 ng/mL) and different concentrations of progesterone. Firefly luciferase activity was normalized to the activity of Renilla luciferase under control of the thymidine kinase promoter. Relative luciferase activity compared with that of the controls is shown. * p < 0.05, ** p < 0.01, and *** p < 0.001 compared with control cells; # p < 0.05 and ## p < 0.01 compared with cells treated with TGF-β1. (**B**) Phosphorylation of endogenous Smad2/3 protein. A549 cells were stimulated with TGF-β1 (10 ng/mL) and progesterone (10 μg/mL) for 1 h. Smad2/3 phosphorylation was detected by immunoblotting using anti-phospho-Smad2/3 (upper lane) or Smad2/3 (lower lane) antibodies. A representative blot from three independent experiments is shown.

To analyze the possible effect of progesterone on Smad signaling in lung epithelial cells, a TGF-β1-sensitive (CAGA)_12_-luciferase construct was transfected into A549 cells. As a positive control, TGF-β1 significantly induced luciferase reporter gene activity compared with that in untreated, transfected A549 cells (12 ± 4-fold, p < 0.001) (Fig 1). Progesterone at different concentrations (0.1 to 20 μg/mL) alone did not activate Smad signaling (Fig 1). At the protein level, no phosphorylation of Smad2/3 induced by progesterone could be detected (Fig 2B).

### Effect of progesterone on TGF-β1-induced Smad signaling in lung epithelial cells

To study the effect of progesterone on TGF-β1-induced Smad signaling in lung epithelial cells, A549 cells were treated with TGF-β1 in the presence of progesterone after transfection with the (CAGA)_12_-luciferase construct. The effect on Smad signaling was measured using luciferase assays and western blotting analysis. Progesterone inhibited TGF-β1-induced reporter gene activity in a concentration-dependent manner (Fig 2A). The highest applied concentration of progesterone (20 μg/mL) reduced TGF-β1-induced Smad activity 0.25 ± 0.15-fold (p = 0.0079) (Fig 2A). To confirm these results at the translational level, TGF-β1 Smad2/3 phosphorylation, the key step for Smad2/3 signaling to the nucleus, was investigated using western blotting with specific anti-phospho-Smad2/3 antibodies. The results showed that progesterone alone had no effect on Smad2/3 phosphorylation, while progesterone inhibited TGF-β1-induced Smad2/3 phosphorylation (anti Smad2/3-P) (Fig 2B). The general abundance of Smad2/3 was neither modified by TGF-β1 nor by progesterone (anti Smad2/3) (Fig 2B).

Taken together, the results suggested that progesterone inhibited the effect of TGF-β1 on the activation of Smad signaling in lung epithelial cells. However, progesterone alone had no effect on Smad signaling.

### Effect of progesterone on TGF-β1-induced CTGF expression in lung epithelial cells

**Fig 3:**
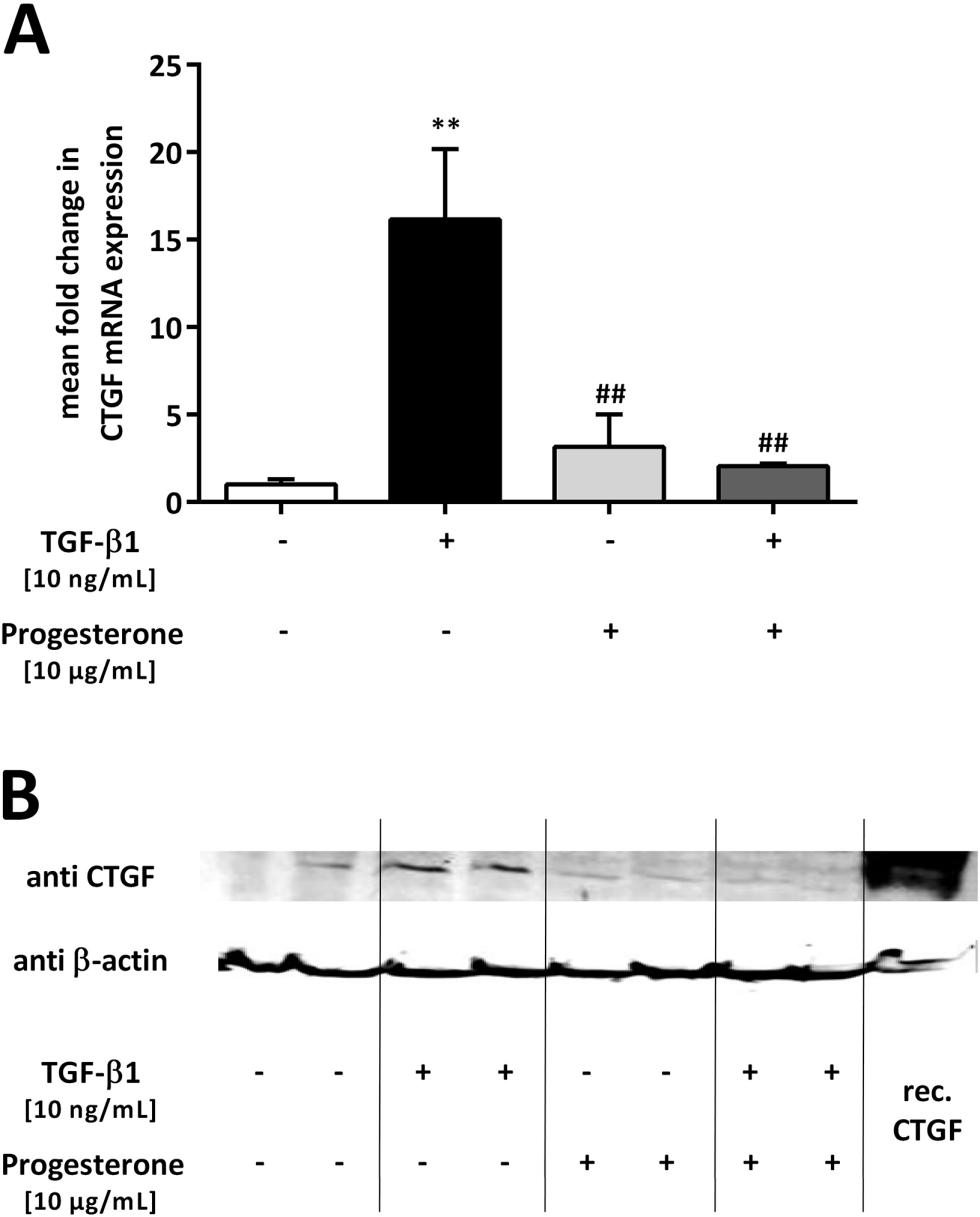
Progesterone inhibits transforming growth factor beta 1 (TGF-β1)-induced connective tissue growth factor (CTGF) expression in lung epithelial cells. A549 cells were incubated with TGF-β1 (10 ng/mL) and progesterone (10 μg/mL). qPCR of *CTGF* mRNA was performed after 12 h (**A**) and western blotting analysis after 24 h (**B**). Relative mRNA levels of *CTGF* were calculated by normalizing signals to detected *GAPDH* mRNA. Differences compared with untreated cells were calculated. Means + SD of at least n = 3 independent experiments are shown. In **B**, a representative immunoblot of CTGF and β-actin of n = 3 independent experiments is shown. ** p < 0.01 compared with control cells; ## p < 0.01 compared with cells treated with TGF-β1.

Next, we investigated the effect of progesterone on the expression of the TGF-β1/Smad-regulated gene *CTGF* in lung epithelial cells. A549 cells were treated with TGF-β1 (10 ng/mL) and progesterone (10 μg/mL), and 12 h later, *CTGF* mRNA expression was measured. Upon single stimulation with TGF-β1, *CTGF* mRNA levels increased by 16.2 ± 4.0-fold compared with that in the untreated cells (p = 0.0021) (Fig 3A), while progesterone had no significant effect on *CTGF* mRNA expression if applied alone (Fig 3A). TGF-β1-induced *CTGF* mRNA expression was reduced 0.13 ± 0.01-fold (p = 0.0055) if the cells were additionally treated with progesterone (Fig 3A). This inhibitory effect of progesterone on TGF-β1-mediated *CTGF* mRNA upregulation was confirmed at the protein level using western blotting analysis (Fig 3B).

These results demonstrated that progesterone abolished TGF-β1-induced *CTGF* expression in A549 cells.

### Effect of progesterone on TGF-β1-induced transgelin expression in lung epithelial cells

**Fig 4:**
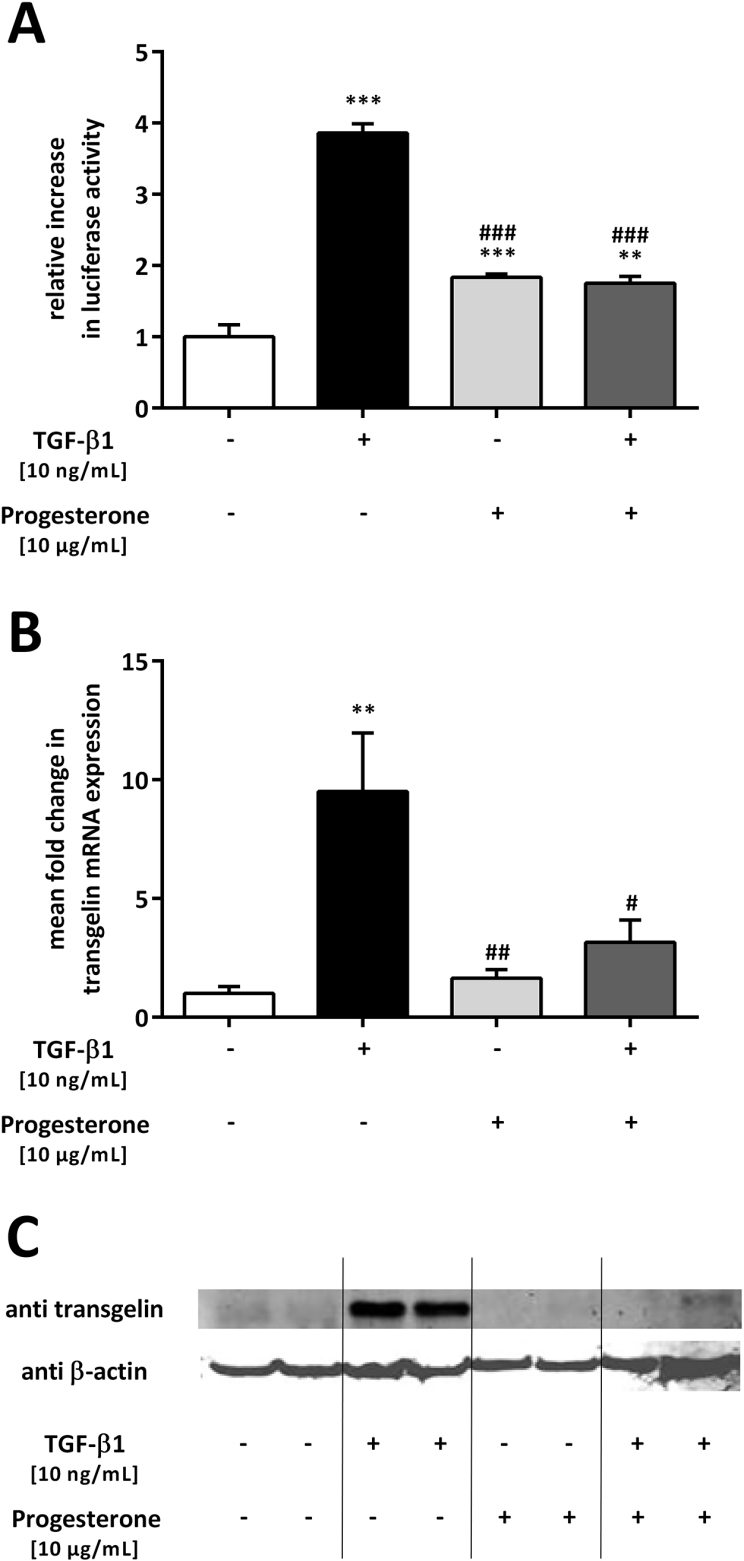
Progesterone inhibits transforming growth factor beta 1 (TGF-β1)-induced transgelin expression in lung epithelial cells. A549 cells were incubated with TGF-β1 (10 ng/mL) and progesterone (10 μg/mL). Promoter analysis (**A**) and qPCR of transgelin (*TAGLN*) mRNA (**B**) were performed after 12 h, and western blotting analysis (**C**) after 72 h. Relative mRNA levels of *TAGLN* were calculated by normalizing signals to detected *GAPDH* mRNA (**B**) and compared with untreated cells. Means + SD of at least n = 3 independent experiments are shown. In **C**, a representative immunoblot of transgelin and β-actin of n = 3 independent experiments is shown. ** p < 0.01 and *** p < 0.001 compared with control cells; # p < 0.05, ## p < 0.01, and ### p < 0.001 compared with cells treated with TGF-β1.

*TAGLN* is another TGF-β1/Smad-regulated gene involved in airway remodeling; therefore, we wanted to examine the effect of progesterone on TGF-β1-induced upregulation of this protein and its mRNA in lung epithelial cells. A549 cells were transfected with a luciferase-reporter construct containing the promoter of human *TAGLN* and subsequently treated with TGF-β1 and progesterone (Fig 4A). In the presence of progesterone, TGF-β1-induced stimulation of *TAGLN* promoter activity was significantly inhibited (p < 0.001) (Fig 4A). To analyze changes in transgelin mRNA and protein expression, A549 cells were treated with TGF-β1 (10 ng/mL) and progesterone (10 μg/mL) and transgelin mRNA and protein levels were measured 12 or 72 h later, respectively. TGF-β1 alone increased *TAGLN* mRNA levels 9.5 ± 2.5-fold compared with that in untreated cells (p = 0.0031) (Fig 4B). In the presence of progesterone, TGF-β1-mediated upregulation of *TAGLN* mRNA expression was diminished 0.33 ± 0.14-fold (p = 0.0245) (Fig 4B). This inhibitory effect was confirmed at the protein level using western blotting analysis (Fig 4C).

Altogether, our data confirm that progesterone inhibited TGF-β1-mediated upregulation of transgelin at the promoter, mRNA, and protein level in lung epithelial cells.

### Effect of progesterone on TGF-β1-induced plasminogen activator inhibitor-1 (PAI-1) expression in lung epithelial cells

**Fig 5:**
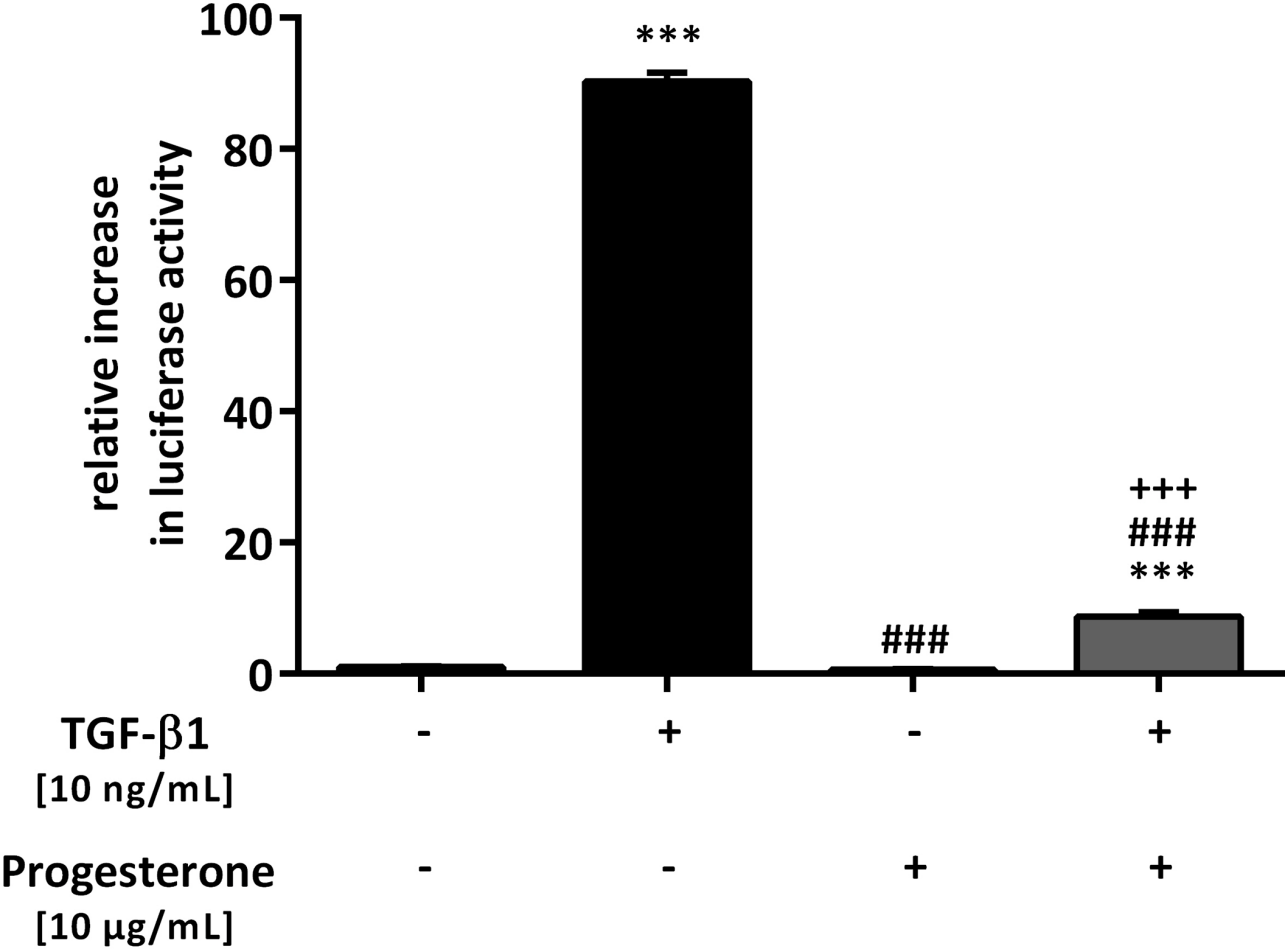
Effect of progesterone on transforming growth factor beta 1 (TGF-β1)-induced plasminogen activator inhibitor-1 (PAI-1) expression in lung epithelial cells. The TGF-β1-sensitive *PAI-1* promoter luciferase reporter construct was stably transfected into mink lung epithelial cells (MLECs). Cells were treated with TGF-β1 (10 ng/mL) and/or progesterone (10 μg/mL). Firefly luciferase activity was normalized to the activity of Renilla luciferase under control of the thymidine kinase promoter. Relative luciferase activity compared with that of the controls is shown. *** p < 0.001 compared with control cells; ### p < 0.001 compared with cells treated with TGF-β1; +++ p < 0.001 compared with cells treated with progesterone.

In addition to CTGF and transgelin, PAI-1 is also regulated via TGF-β1/Smad and is involved in airway remodeling. Using stably transfected MLECs bearing an expression construct containing a truncated *PAI-1* promoter fused to the firefly luciferase reporter gene, the effect of progesterone on TGF-β1-induced *PAI-1* promoter activity was studied [18]. TGF-β1 induced a 90.3 ± 1.4-fold increase in *PAI-1* promoter activity (p < 0.001), which was significantly reduced by the additional presence of progesterone (p < 0.001). Progesterone alone had no effect (Fig 5).

## Discussion

The potential consequences of exogenous, prenatal progesterone application on the lung function of preterm infants are poorly defined. Progesterone receptors are expressed in the lung; therefore, it is likely that progesterone is also involved in fetal lung development and in the pathogenesis of BPD [22]. In newborn piglets, combined progesterone and estradiol antagonism during pregnancy decreased lung alveolarization [23]. In this study, we focused on the possible influence of progesterone on Smad signaling, a key pathway involved in BPD, in lung epithelial cells.

During mid and late gestation, the human fetus is usually exposed to high amounts of progesterone, which is produced by the placenta [4, 24] and then delivered into the circulation, leading to 100-fold elevated concentrations in the fetus compared with the non-pregnant status [24, 25]. Plasma levels range between 0.64 and 5.4 nmol/mL in the umbilical vein and between 0.32 and 3.8 nmol/mL in the umbilical artery [24, 26, 27]. Delivery stops the placental supply and as a consequence, progesterone levels drop by about 100 times within a few hours after birth [25, 28, 29]. Hence, lung development of term infants *in utero* usually takes place in a milieu of high concentrations of progesterone and especially during fetal life, high local levels can be found in the fetal lung, which decrease drastically after birth [30]. While this is a physiological condition for the term infant, an extremely preterm infant is disconnected from the supply of these hormones at a much earlier developmental stage, which has possible consequences for the development and function of the innate lung; i.e., the extrauterine lung develops in a milieu with much lower progesterone concentrations compared with that of term infants [3, 4, 31]. In early clinical trials, the replacement of estradiol together with progesterone in extremely preterm infants to mimic high *in utero* levels of 17ß-estradiol and progesterone was associated with a trend towards a reduced incidence of BPD [3, 4].

In addition to this endogenous change of progesterone following preterm delivery, the exogenous administration of progesterone during pregnancy may influence progesterone levels in preterm infants. Clinical studies showed that weekly administration of progesterone to pregnant women could reduce the rate of preterm delivery in high-risk patients and reduced the incidence of complications in newborns [32–34]. The applied progesterone passes the placenta into the fetal circulation, thereby enhancing levels in the fetus [33]. Data on human serum concentrations administered in pregnancy, which further elevate high levels of progesterone during pregnancy, are lacking, and the optimal dosing and concentrations of progesterone to prevent preterm delivery are currently unknown [35].

The molecular mechanism by which progesterone influences lung function is unclear. While there are only a few *in vitro* studies on the effect of sole progesterone on different lung functions, more studies have focused on the combined application of progesterone and estradiol. Only this combined application enhances vascular endothelial growth factor (VEGF) and surfactant protein (SP) expression in lung cells [36]. In addition, it could be shown that prenatal estradiol and progesterone deprivation impaired alveolar formation and amiloride-sensitive alveolar fluid clearance [23]. Furthermore, a possible effect of progesterone on inactivation of prostaglandin E2 in the lung has been described [37]. Only progesterone, and not a combination of estradiol and progesterone, is administered during pregnancy to prevent preterm delivery; therefore, we exclusively focused on the effect of progesterone in this study.

Progesterone receptors are detectable on the surface of A549 cells [38]; for that reason, we first studied the potential direct effect of progesterone on Smad signaling, while several studies have focused on the effect of the two other sex hormones, estrogen and dihydrotestosterone (DHT). Estrogen can inhibit TGF-β signaling by promoting Smad2/3 degradation [39]. These data support the concept that combined replacement of estrogen and progesterone in preterm infants acts synergistically on Smad signaling and therefore may help to prevent BPD. In addition, DHT also suppresses transcriptional responses of TGF-β by impeding the binding of Smad3 to the SBE (Smad binding element) [40]. In the present study, progesterone alone showed no effect on Smad signaling; however, progesterone was able to antagonize TGF-β-induced Smad activation in a dose-dependent manner. TGF-β and Smad signaling participate in the pathogenesis of BPD as important regulatory factors during pulmonary vascular development and alveolarization [41–43]. Thus, progesterone may also act as a protective factor for the development of BPD by inhibiting the Smad signaling pathway.

In addition, we were able to demonstrate that progesterone inhibited the expression of the TGF-β1/Smad-regulated genes *CTGF*, *TAGLN*, and *PAI-1* in lung epithelial cells. In mice, uterine estradiol and to a lower extend also progesterone alone stimulated CTGF expression, and the stimulatory effect of estradiol on expression of CTGF could be antagonized in the presence of progesterone [44]. This finding is in agreement with our results, which showed antagonizing effects of TGF-β-induced *CTGF*, *TAGLN*, and *PAI-1* expression by progesterone. Dysregulation of CTGF, transgelin, and PAI-1 has been implicated in the pathogenesis of BPD [10–13].

The levels of progesterone used in this study appear to be above the physiological plasma levels observed during pregnancy [36]. However, it is believed that under *in vitro* conditions, higher steroid levels are necessary to yield cellular effects [36]. In earlier *in vivo* studies, these high concentrations were applied to generate physiological effects [45, 46]. Another interesting aspect is that tissues can intrinsically produce steroid hormones, which may then contribute to higher local tissue concentrations in combination with plasma steroids [36]. Thus, in rodent hippocampal tissue, local estrogen production yields tissue steroid levels at approximately 10^−9^ M, which is above plasma concentrations [47]. Data on human serum concentrations of progesterone administered in pregnancy, which further elevate levels during pregnancy, are currently lacking [35]. For this reason, the high levels of progesterone used during this study may have clinical applicability [35].

In summary, the inhibitory effect of progesterone on TGF-β1-induced Smad signaling and its regulated genes described in the present study may attenuate the development of BPD. Future studies will help to identify underlying molecular mechanisms in greater detail and to verify the observations in animal models *in vivo*.
